# Deworming in non-pregnant adolescent girls and adult women: a systematic review and meta-analysis

**DOI:** 10.1186/s13643-018-0859-6

**Published:** 2018-12-20

**Authors:** Elizabeth Tanjong Ghogomu, Shalini Suresh, Pura Rayco-Solon, Alomgir Hossain, Jessie McGowan, Juan Pablo Peña-Rosas, Vivian Welch

**Affiliations:** 1 Bruyère Research Institute, Bruyère, 310 - 85 Primrose Avenue E, Ottawa, ON K1R 7G5 Canada; 2 Bruyère Research Institute, Bruyère , 312 - 85 Primrose Avenue E, Ottawa, ON K1R 7G5 Canada; 3 Bruyère Research Institute, Bruyère, 305 - 85 Primrose Avenue E, Ottawa, ON K1R 7G5 Canada; 4 Department of Nutrition for Health and Development, World Health Organization, 20 Avenue Appia, CH-1211, Geneva 27, Switzerland; 5 Cardiovascular Research Methods Centre, University of Ottawa Heart Institute, Ottawa, ON K1Y 4W7 Canada; 6 School of Epidemiology and Public Health, University of Ottawa, 600 Peter Morand Crescent, Ottawa, ON K1G 5Z3 Canada

**Keywords:** Deworming, Soil-transmitted helminthiasis, Women, Non-pregnant, Anaemia, Haemoglobin

## Abstract

**Background:**

The impact of deworming on parasite load, nutritional status and other health outcomes of non-pregnant adolescent girls and adult women is uncertain.

**Methods:**

MEDLINE, EMBASE, CINAHL, the Cochrane Central Register of Controlled Trials, the WHO International Clinical Trials Registry Platform, the Cochrane Database of Systematic Reviews and Food and Technology Abstracts databases were searched until 24 September 2018. Studies were included if they were randomised controlled trials (RCTs), controlled before and after studies or interrupted time studies comparing deworming with no intervention or placebo in non-pregnant adolescent girls and women 10 to 49 years old. Outcomes of interest included parasite load, reinfection, anaemia, severe anaemia, iron deficiency, diarrhoea or all-cause morbidity. Risk of bias was assessed using the Cochrane risk of bias tool.

**Results:**

We included four RCTs of mass deworming involving 1086 participants, in the analyses. Mass deworming probably reduces the prevalence of roundworm infection (RR 0.29; 95% CI 0.14 to 0.62; 2 trials; 1498 participants, moderate certainty evidence), prevalence of hookworm infection (RR 0.32; 95% CI 0.18 to 0.59; 2 trials; 1498 participants, moderate certainty evidence), prevalence of whipworm infection (RR 0.77; 95% CI 0.65 to 0.91; 2 trials; 1498 participants, moderate certainty evidence) compared to the control group. Deworming may make little or no difference in prevalence of anaemia (RR 0.82; 95% CI 0.60 to 1.11, 3 studies, 683 participants, low certainty evidence) and prevalence of iron-deficiency (RR 0.89; 95% CI 0.64 to 1.23, 1 study, 186 participants, low certainty evidence) compared to control. We are uncertain whether deworming reduces the prevalence of severe anaemia compared to control as the certainty of evidence was very low. None of the included studies assessed screen and treat deworming or reported reinfection, diarrhoea or adverse events.

**Conclusions:**

Mass deworming probably reduces the prevalence of soil-transmitted helminth infections but may have little or no effect on anaemia and iron-deficiency in adolescent girls and non-pregnant women in comparison to no intervention or placebo. We are uncertain about the effect on severe anaemia. These results are limited by sparse data and the moderate to very low quality of evidence available.

**Systematic review registration:**

The protocol was registered in PROSPERO (registration number: CRD42016039557).

Primary source of funding: Evidence and Programme Guidance unit, Department of Nutrition for Health and Development, World Health Organization (WHO).

**Electronic supplementary material:**

The online version of this article (10.1186/s13643-018-0859-6) contains supplementary material, which is available to authorized users.

## Background

Soil-transmitted helminthiasis (STH) affects 24% (2 billion people) of the global population [1]. The burden of disease of STH was estimated at 5.18 million disability adjusted life years DALYs globally in 2010 [2]. These infections rarely cause death, and therefore the burden is predominantly due to morbidity. The most common species of soil-transmitted helminths that infect people are the roundworm (*Ascaris lumbricoides*), hookworms (*Necator americanus* and *Ancylostoma duodenale*) and the whipworm (*Trichuris trichiura*). Roundworms cause intestinal obstruction and biliary or pancreatic disturbances [2, 3]. STH may thus lead to iron deficiency, anaemia, vitamin A deficiency or other nutritional impairments [1]. Hookworms bite into intestinal mucosa, secrete anticoagulants and feed on blood [4–9]. Whipworms can impair fat digestion and cause vitamin malabsorption, blood loss and appetite suppression in intense disease, through a disease mechanism known as trichuriasis syndrome [10–14]. Anthelminthic drugs such as albendazole, mebendazole, levamisole, pyrantel, piperazine and thiabendazole are administered as pharmacological deworming interventions for individuals with soil-transmitted helminth infections [15, 16]. The World Health Organization (WHO) recommends these deworming treatments to be administered periodically to all at-risk populations (preschool age children, school age children, women of childbearing age and pregnant women in the second or third trimester) once a year in areas with 20% to 50% soil-transmitted helminth prevalence^1^ and twice a year in areas with greater than 50% helminth prevalence, without previous individual diagnosis [17–20]. This is often referred to as mass deworming. These actions should be complemented by simultaneous implementation of plans to improve sanitation and hygiene and to supply adequate safe water to the community [17–20].

Deworming treatments aim to reduce the intensity of helminth infection, to protect infected individuals and prevent further transmission [17, 21]. Anthelminthic drugs differentially reduce worm burden and in turn can reduce the morbidity associated with STH. Cure rates are highest for roundworms, lower for hookworms and very low for whipworms [22]. With many deworming treatment programs taking place in community-based settings, there is the potential to also see a spill-over effect of decreased worm burden among untreated people and their household members [23]. This occurs because there is a lower risk of untreated people contracting the disease from treated people.

Menstruating adolescent girls and women are regarded as an at-risk subpopulation by WHO [1]. The monthly blood loss due to menstruation leads to rapid iron depletion, an essential compound of red blood cells. Similarly, the period of rapid growth, expansion of red cell mass and increased tissue requirements in adolescent girls lead to iron depletion [24]. STH infections could further increase their risk for iron deficiency and anaemia, therefore resulting in a poorer overall nutritional status [1]. Hence, it is important to know whether deworming, and more specifically mass deworming can reduce the parasite load, improve the nutritional status and other health outcomes of menstruating adolescent girls and adult women living in areas endemic for soil-transmitted helminths.

This systematic review and meta-analysis aims to evaluate the effects and harms of regular deworming in areas endemic for soil-transmitted helminths among all non-pregnant adolescent girls and women between the ages of 10 and 49 years. The outcomes of interest are parasite load (defined here as prevalence of soil-transmitted helminthiasis among sample population), anaemia, severe anaemia, iron deficiency, diarrhoea, reinfection rate and all-cause morbidity.

## Methods

This review was conducted based on a pre-planned protocol registered at PROSPERO CRD42016039557, attached in Additional file 1. It is reported following the PRISMA statement [25]. The PRISMA checklist is in Additional file 2.

### Inclusion criteria

#### Study design

We included randomised controlled trials, which may be randomised at the individual or cluster level. We considered quasi-experimental studies such as controlled before and after studies and interrupted time series (with at least three time points before and after the intervention). We also considered studies with a post-only measurement, providing the baseline groups are considered comparable on potential confounders such as socioeconomic status, level of education, worm and anaemia prevalence.

We restricted study durations to a minimum of 4 months as shorter studies would not have an adequate time to observe substantive change in the primary outcomes.

#### Population

We considered non-pregnant women between the ages of and including 10 to 49. We included studies that reported results for women in the above age groups, disaggregated from other populations. If a study included this population, but data was not reported separately from women aged 10–49 years, this study was classified as a pending study and the authors were contacted for age and sex-stratified data.

#### Interventions and comparators

Interventions were deworming treatments targeted at soil-transmitted helminth infections. Mass deworming and screen-to-treat interventions were eligible for inclusion. The WHO Model List of Essential Medicines [16] was consulted to select the following anthelmintics as included interventions: albendazole, mebendazole, pyrantel, piperazine, levamisole, thiabendazole. The comparators assessed were no intervention or a placebo. Studies that included deworming in all treatment arms were excluded. Other interventions were excluded unless present in both treatment and control arms. For example, concomitant iron was allowed as long as it was provided in both the treatment and control groups.

#### Outcomes

The outcomes outlined below were selected for the review.

##### Primary outcomes

1. Parasite load (defined as the prevalence of helminth infection in the study population)
2. Anaemia (defined as haemoglobin concentration of less than 120 g/L [22] for non-pregnant women, adjusted by smoking and altitude, where appropriate)
3. Severe anaemia (defined as haemoglobin concentration lower than 80 g/L [22] adjusted by smoking and altitude, where appropriate)
4. Iron deficiency (as defined by trialists)
5. Diarrhoea (three liquid stools or more per day)
6. All-cause morbidity (number of patients with at least one episode of any disease during the study period)
7. Any adverse effects (any, as defined by trialists)

##### Secondary outcomes

1. Physical function/work capacity
2. Reinfection (as defined by trialists)

### Search methods for identification of studies

In consultation with a librarian scientist (JM), we developed a search strategy in MEDLINE, which was then translated into the other database formats. A modified Effective Practice and Organisation of Care (EPOC) study design filter and a systematic review filter were used to identify relevant study designs [26]. All database searches were conducted in OVID, except CINAHL (EBSCO Host). The searches were conducted from the earliest dates of the databases to 24 September, 2018. We did not apply any language restrictions.

#### Electronic searches

The search includes the following health and non-health electronic databases: MEDLINE, CINAHL, EMBASE, the Cochrane Central Register of Controlled Trials, the Cochrane Database of Systematic Reviews and Food Science and Technology Abstracts up to 24 September, 2018. The search strategies for these databases can be found in Additional file 3.

#### Additional search strategy

We searched reference lists of included studies and relevant systematic reviews, including a review of deworming for children [27] and a published review of deworming for non-pregnant populations [28] to identify potential studies. We also searched the WHO International Clinical Trials Registry Platform and ClinicalTrials.gov to identify potentially relevant ongoing trials.

We contacted authors of studies that met our eligibility criteria but did not provide disaggregated data for our population of interest or relevant data for our outcomes of interest.

### Data collection and analysis

#### Selection of studies

Two reviewers (VW, SS) independently screened titles and abstracts based on the inclusion criteria, in order to address the following questions: (a) Does the intervention include pharmacologic deworming treatment which is provided by mass or targeted administration to an identified high-risk group? (b) Is at least one of the following outcomes measured: anaemia, iron deficiency, diarrhoea, severe anaemia, reinfection, all-cause morbidity? (c) Does the population include women between the ages of 10 and 49 years? (d) Is the length of time from intervention to follow-up 4 months or longer? (e) Does the study design include an appropriate comparison group (i.e. control group or pre-post or post-only if baseline characteristics similar)?

Two authors (VW, SS) pre-tested the title and abstracts screening questions. If any one of these questions was answered as ‘no’, then the study was excluded from further consideration.

If all questions were answered as ‘yes’ or ‘unclear’, then the study was included for full-text screening. Coding for screening was entered into a systematic review software manager, Covidence [29]. After each reviewer independently screened studies, any discrepancies around decisions for inclusion or exclusion were discussed and reconciled accordingly with a third author (EG). Full texts were retrieved for titles and abstracts accepted for inclusion after discussion by both reviewers. The full text was assessed by two reviewers (VW, SS) for inclusion according to the pre-specified eligibility criteria. Any disagreements were settled by discussion with a third author (EG) who reviewed the full text and decided whether it met the inclusion criteria. For judgments related to appropriate control for confounders in studies with post-only measurements, we planned to consult with a statistician, but this was not needed since none of these study designs were identified.

### Data extraction and management

Two reviewers conducted independent data extraction and risk of bias assessment of all included studies, using an adapted pre-tested EPOC data collection form (see Additional file 4). Information to be extracted included the following:

- Data on study design, details about the participants (including the number in each group), setting (e.g. endemicity, sanitation), intervention (e.g. type of drugs, dose, frequency and process of implementation such as method of delivery—provision of deworming integrated with other programs, amount of supervision), comparison, outcomes (including cost-effectiveness and whether outcomes are validated).
- Data about socio-demographic variables associated with disadvantage, across factors described by the acronym PROGRESS-Plus (Place of residence, Race/ethnicity, Occupation, Gender/sex, Religion, Education, Socioeconomic status and Social capital) [30].
- Data on any effect modifier analyses (e.g. subgroup analyses and meta-regression) conducted in the primary studies.

We compared the extraction by both reviewers (VS, SS) and reached consensus by discussion and consultation with a third reviewer (EG), when necessary.

Authors who reported non-disaggregated data were contacted for more detailed information. Only the studies of authors who provided disaggregated data were included in the analysis.

### Risk of bias in individual studies

We used the Cochrane Risk of Bias tool to assess potential sources of bias in the included randomised controlled studies [31]. The main categories of bias that were assessed are selection bias, performance bias, detection bias, attrition bias and outcome reporting bias.

Since we also considered controlled before-after studies, interrupted time series and post-only studies, we planned to use EPOC’s suggested risk of bias criteria. For controlled before-after studies, we planned to additionally assess baseline imbalance, similarity of outcome measurements and the level of protection against contamination in each study. For interrupted time series studies, we planned to assess the dependence of the intervention on other changes, pre-specification of the shape of the intervention, and the likelihood of the intervention to affect data collection [32].

Risk of bias was assessed for each outcome in each study by two independent reviewers as low, high or unclear risk, with justifications. Consensus was reached between the reviewers.

### Measures of treatment effect

The effect sizes of the continuous outcomes were analysed as weighted mean differences of change scores as these were measured using the same units across studies. Dichotomous outcomes were analysed as risk ratios.

### Unit of analysis issues

Where the unit of allocation is by groups (e.g. schools, communities, village, region), we planned to use the standard deviation adjusted for clustering, if provided by the study. If the study did not adjust for clustering, we would adjust the standard deviations using the variance inflation factor, as described in the Cochrane Handbook [31].

There were no unit of analysis issues with the included studies. For studies with multiple intervention groups, we only considered the groups with the relevant interventions.

### Dealing with missing data

We (VW, SS) collected complete data on items in the data extraction form. If standard deviation or standard error was not provided, we calculated it from other information provided such as exact *p* values, *F* tests or ranges, using formulae in the Cochrane Handbook [31]. We did not impute missing values (e.g. missing variance or outcome data).

### Assessment of heterogeneity

Heterogeneity was assessed by visual inspection of forest plots, chi-squared test and *I*^2^ statistic. *I*^2^ was used to quantify inconsistency across studies, as it describes the percentage of variability in effect estimates that is due to heterogeneity [33]. If there was substantial heterogeneity according to any of these methods, we planned to interpret results in the context of characteristics of the setting and population, as described below.

### Assessment of reporting biases

We planned to assess reporting bias using a funnel plot if there were enough included studies to conduct a plot (> 10 studies).

### Data synthesis

The statistical analysis was done using Review Manager software [34]. We used a random-effects model in order to take into account the effect of varying contexts, populations and settings on the treatment effect. We reported analyses for each outcome and follow-up period separately.

The magnitude of effect and quality of evidence is presented in a summary of findings table (Table 2) with seven primary outcomes. Quality of the evidence was assessed by the GRADE approach, and the summary of findings table was created using the GRADEpro software [35].

### Subgroup analysis and investigation of heterogeneity

We did not plan any subgroup analyses as we expected to have insufficient studies for such analyses. However, we planned to consider variations in the following characteristics between and within studies in interpreting effects:

- Baseline prevalence of any soil-transmitted helminth in the trial, using the cut-offs from the WHO guidelines [18]: less than 20%, 20 to 49%, 50% or higher, unknown/not reported
- Baseline anaemia prevalence in the trial: anaemic (haemoglobin < 120 g/L), non-anaemic, mixed/not reported. Classification of anaemia prevalence according to proportion of anaemic cases in population: severe ≥ 40%; moderate = 20.0–39.9%; mild = 5.0–19.9%; normal ≤ 4.9% [27].
- Class of intensity of infection for individual helminths [36]:
  1. Light-intensity infections: 1–4999 eggs per gram of faeces (epg) in *A. lumbricoides*; 1–999 epg in *T*. *trichiura*; 1–1999 epg in hookworms
  2. Moderate-intensity infections: 5000–49,999 epg in *A*. *lumbricoides*; 1000–9999 epg in *T*. *trichiura*; 2000–3999 epg in hookworms
  3. Heavy-intensity infections: ≥ 50,000 epg in *A*. *lumbricoides*; ≥ 10,000 epg in *T*. *trichiura*; ≥ 4000 epg in hookworms
- Screened for infection: yes/no (i.e. studies with screening as an eligibility criteria such that the entire study population is infected)
- Eligibility criteria that restricts to less severely affected groups such as exclusion of participants with severe anaemia

### Sensitivity analysis

We planned to conduct sensitivity analyses to assess the impact of outlier individual studies (e.g. including only very large studies with > 100 participants, very large effects, very precise confidence intervals) on the overall effect size, as well as risk of bias (including only studies with low risk of bias), treatment compliance and imputed variance inflation factors.

## Results

### Differences from protocol

We modified the lower limit for age in our eligibility criteria from 12 years of age to 10 years of age because the age of adolescence was reduced [37, 38]. We used the WHO measures for severe anaemia, defined by haemoglobin levels < 80 g/L instead of < 70 g/L [22] as stated in the protocol. We decided to add adverse events to our list of primary outcomes (instead of secondary) and we changed reinfection rate to a secondary outcome.

### Results of the search

**Fig. 1:**
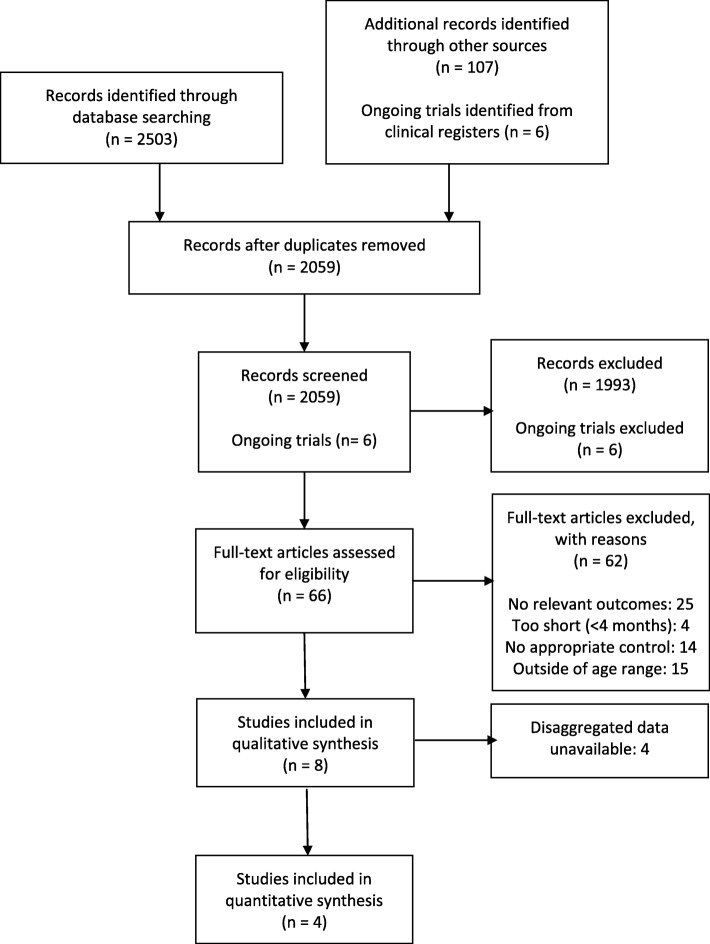
PRISMA flow diagram

The search strategy identified 2295 records including 8 ongoing trials after duplicates were removed. We further reviewed 77 articles in full text and included 4 studies. The PRISMA flow diagram for study selection is shown in Fig. 1.

### Excluded studies

We excluded 67 studies [39–97] because of durations less than 4 months (4 studies), populations of children younger than 10 years of age (16 studies), lack of relevant outcomes reported (25 studies), mixed interventions with other drugs such as praziquantel and lack of an appropriate control group for comparison (16 studies).

Six studies [7, 98–102] were excluded from the analyses because of unavailable disaggregated data and comprised 8008 male and female participants aged 4 years and older.

These studies have been listed in Additional file 5.

### Included studies

We identified 4 randomised controlled trials in 5 publications with a total of 6144 participants that met all the inclusion criteria: Gilgen et al. [103, 104], Gopaldas et al. [105], Miguel et al. [23] and Olds et al. [83]. Within these study populations, the number of participants that matched our population of interest (adolescent girls ≥ 10 years of age and non-pregnant adult women) was 955. These included studies were from Bangladesh [103, 104], India [105], Kenya only [23] and one study was a multi-site trial in China, Philippines and Kenya [83]. Detailed characteristics of these studies are described in Table 1.

**Table 1:** Baseline sample characteristics of included studies (all participants)

Author, year	Sample size	Country	Age (range in years)	Intervention arms	Comparisons in analyses	Study length (months)	Baseline anaemia (%)	Parasite prevalence (%)	Parasite intensity (eggs per gram)
Gilgen, 2001 [103, 104]	553	Bangladesh	14–66	1. 200 mg ferrous fumarate + 200 mg folic acid, 1×/week 2. 400 mg albendazole single dose at week 1 and week 12 3. Interventions 1 + 2 4. Placebo	2 vs. 4 3 vs. 1^a^	5.5	85.7	Ascaris: 47.6 Hookworm: 74.4 Trichuris: 56.8	Ascaris: 20.8; Hookworm: 57.7; Trichuris: 14.8
Gopaldas 1983 [105]	170	India	5–13	1. Placebo 2. Anthelmintic + antiprotozoal 3. Anthelmintic + iron 4. Anthelmintic + iron + vitamin A 5. Iron Anthelmintic: mebendazole 100 mg, 2×/day (3 consecutive days, 2×/4 months Antiprotozoal: tinidazole 50 mg/kg body weight for 3 days consecutively Iron: iron-folic acid (20 mg elemental iron and l00 mcg folic acid), 1×/day for 60 days, repeated at 4 months Vitamin A: one dose of 200,000 I.U.)	3 vs. 5^a^	8	93.0	Any helminth: 11%	NR
Olds 1999 [83]	1518	Philippines, China, Kenya	5–18	1. Placebo (physically identical) 2. Albendazole (single dose 400 mg) 3. Praziquantel (one dose of 40 mg/kg body weight) 4. Albendazole + praziquantel	2 vs. 1	6	75.2	Ascaris: 60.2 Hookworm: 52.1 Trichuris: 81.0	Generally light infections
Miguel 2004 [23]	3903	Kenya	6–18	1. Albendazole, 400 mg, 2×/year 2. Albendazole, 600 mg, 2×/year + praziquantel 40 mg/kg, 2×/year 3. No treatment	1 vs 3	24	4.0	Ascaris: 42.0 Hookworm: 77.0 Trichuris: 55.0	Ascaris: 2337 ± 5156; Hookworm: 426 ± 1055; Trichuris: 161 ± 470

^a^Secondary analyses

In the study in Bangladesh, women aged 14–66 years, who worked as full-time tea pluckers in a tea estate in Bangladesh were recruited to join the study. They were randomly assigned to one of four intervention groups. We used group 2 vs. 4 for our primary analysis, and we included group 1 vs. group 3 as a secondary analysis of deworming + iron vs. iron alone (see Table 1). The authors reported outcomes of haemoglobin levels, labour productivity, parasite loads, ferritin levels and anthropometry and were contacted for additional data on prevalence of anaemia, iron deficiency and severe anaemia [103, 104].

The study based in India was among school girls aged 5–13 years from low-income families in Baroda, India. The authors reported data separately for girls aged 5–9 and 10–13. Only the data from the 10–13 years subgroup was used for our analyses. At baseline, this population had helminth infection prevalence of 11% and anaemia prevalence of 93% [105]. There were five intervention groups (see Table 1). The authors reported outcomes of haemoglobin, feasibility and efficiency of treatment administration and prevalence of conjunctival xerosis [105]. None of the intervention arms met our criteria for assessing deworming alone compared to placebo; however, group 3 vs. 5 was analysed with Gilgen et al. [104] in the secondary analysis to assess the effect of deworming + iron vs. iron alone.

The study in Kenya only was a cluster-randomised trial conducted among school children including males and females, aged 6–18. Participants were randomised to interventions at the level of the school. At baseline, this population had STH prevalence of 92%, whereas the overall proportion of anaemia was unreported. The included children were assigned to one of three groups described in Table 1. The authors reported outcomes of weight, height and anaemia. Additional disaggregated data for ≥ 10-year-old females (*n* = 132 for deworming with albendazole only and *n* = 228 for no intervention/control) in the study on anaemia and parasite load was retrieved from individual participant data provided by the authors and included in the analyses [23].

The multi-site trial was conducted in the Philippines, China and Kenya and included males and females aged 5–18. The baseline STH prevalence in this population was between 50 and 80% and the baseline anaemia prevalence was 75.2%. Participants were randomly assigned to one of four intervention arms (see Table 1). The authors reported outcomes of infection status, growth parameters and haemoglobin. Additional disaggregated data for ≥ 10-year-old females (*n* = 27 for deworming and *n* = 24 for no intervention/control) in the study on anaemia and severe anaemia was retrieved from the authors and included in the analyses [83].

In these studies, pregnancy was not explicitly ruled out by eliciting a menstrual history. In one included study [83], pregnancy was ruled out by questioning the participants about possible pregnancy risk and excluding as necessary. One study [23] only included girls under the age of 13 to potentially rule out the possibility of pregnancy, but did not specifically verify pregnancy status. One study [104] reported that only non-pregnant women were enrolled in the study while one study [105] did not report verification of pregnancy status.

### Additional data from authors

We contacted authors for disaggregated data on females for our outcomes of interest and we received data from 3 studies [23, 83, 105] which included 683 women, and none for 6 studies [7, 98–102] (Bhoite et al. 2012 [98]; de Ruiter et al. 2017 [101]).

### Risk of bias assessment

**Fig. 2:**
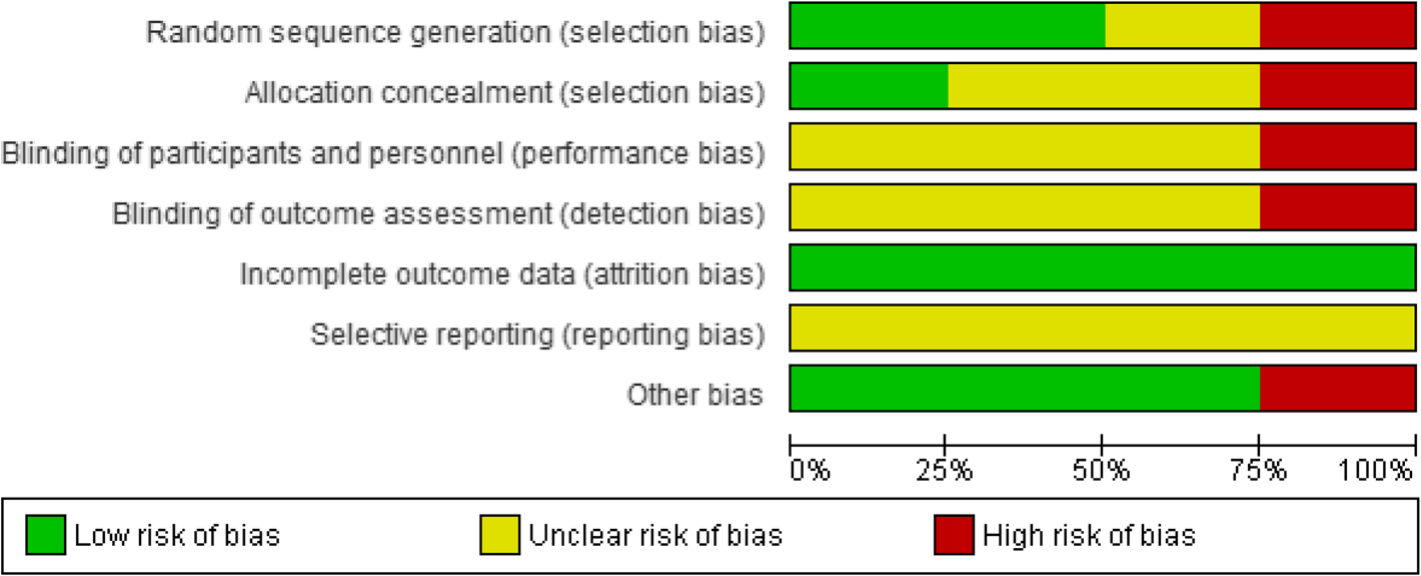
Risk of bias summary for included studies

**Fig. 3:**
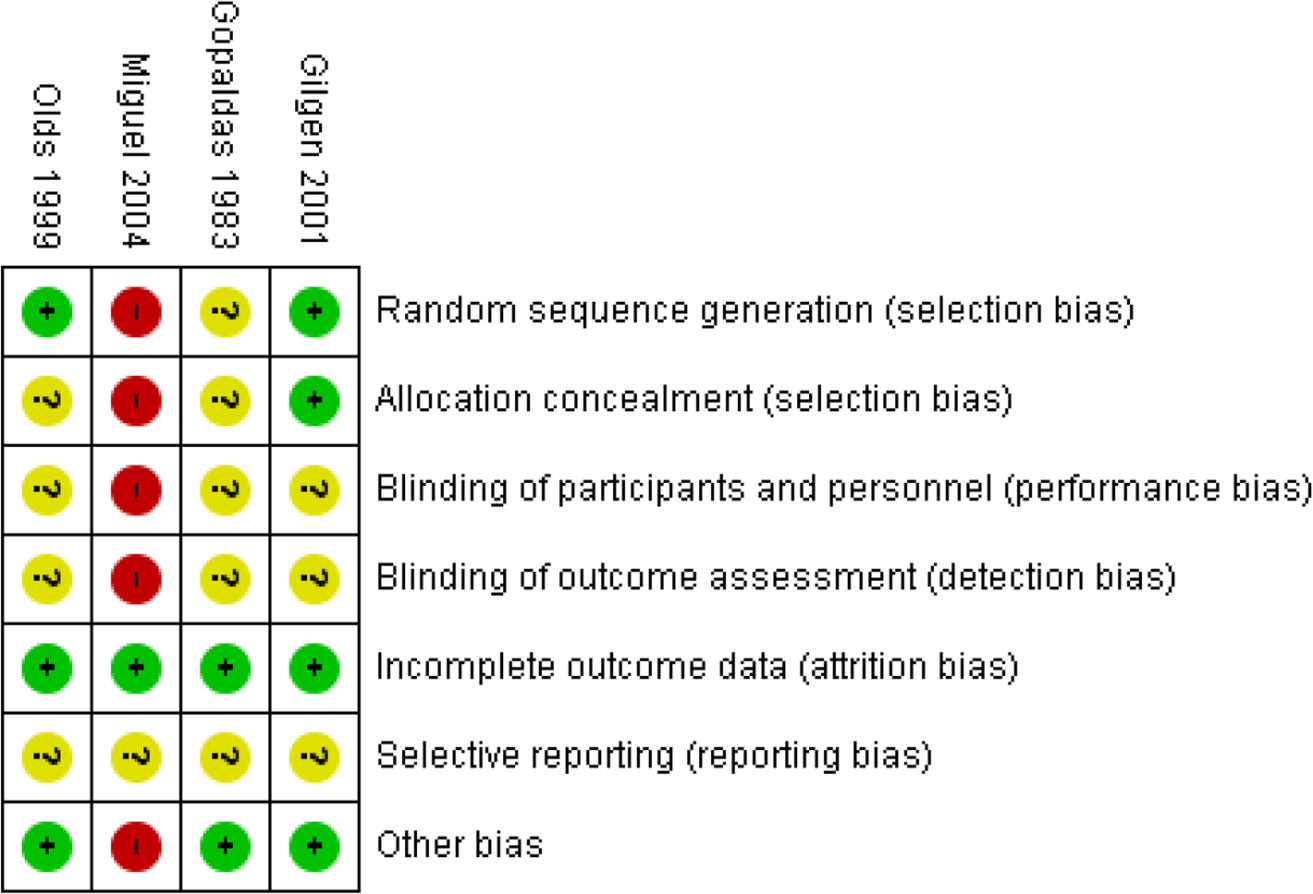
Forest plot for end of study anaemia prevalence as mean difference

The risk of bias for the included studies is shown in Figs. 2 and 3.

Allocation (selection bias): Gilgen et al. [104] had a low risk of bias for sequence generation and allocation concealment, whereas Olds et al. [83] and Gopaldas et al. [105] had an unclear risk of selection bias. Miguel et al. [23] had a high risk of selection bias because of inadequate sample and cluster selection methods.

Blinding (performance bias and detection bias): there was unclear risk of performance bias (blinding of participants and personnel) and detection bias (blinding of outcome assessors) in Gilgen et al. [104], Gopaldas et al. [105] and Olds et al. [83]. There was a high risk of performance bias in Miguel et al. [23] as allocation methods were not concealed due to which personnel may have known intervention assignments.

Incomplete outcome data (attrition bias): there was low risk of attrition bias in all the included studies.

Selective reporting (reporting bias): reporting bias was unclear in all four included studies.

Other potential sources of bias: there was low risk of other sources of bias in all included studies except Miguel et al. [23] which had a high risk of other sources of bias in due to baseline imbalances.

### Effects of interventions

The primary comparison of interest was mass deworming vs. no intervention/placebo. Three trials were included in this comparison [23, 83, 104]. The summary of findings for this comparison is detailed in Table 2.

**Table 2:** Summary of findings

Patient or population: non-pregnant women aged 10–66 Setting: STH endemic areas Intervention: deworming Comparison: no intervention/placebo						
Outcomes	Anticipated absolute effects^a^ (95% CI)		Relative effect (95% CI)	№ of participants (studies)	Quality of the evidence (GRADE)	Comments
	Risk with control	Risk with deworming				
Anaemia prevalence assessed with haemoglobin levels < 120 g/L Follow up: mean 6 months	398 per 1000	327 per 1000 (239 to 442)	RR 0.82 (0.60 to 1.11)	683 (3)	⨁⨁◯◯ Low^b,c^	
Iron deficiency prevalence assessed with ferritin levels < 12 μg/L Follow up: mean 6 months	464 per 1000	413 per 1000 (297 to 571)	RR 0.89 (0.64 to 1.23)	186 (1)	⨁⨁◯◯ Low^b,c^	
Severe anaemia			RR 6.25 (0.34 to 115.15)	51 (1)	⨁◯◯◯ Very low^b,d^	
Parasite load—Ascaris assessed with: prevalence follow up: 6 months	327 per 1000	95 per 1000 (46 to 202)	RR 0.29 (0.14 to 0.62)	1498 (2)	⨁⨁⨁◯ Moderate^b^	
Parasite load—Hookworm assessed with: prevalence follow up: 6 months	331 per 1000	106 per 1000 (60 to 195)	RR 0.32 (0.18 to 0.59)	1498 (2)	⨁⨁⨁◯ Moderate^b^	
Parasite load – Trichuris assessed with: prevalence follow up: 6 months	277 per 1000	213 per 1000 (180 to 252)	RR 0.77 (0.65 to 0.91)	1498 (2)	⨁⨁⨁◯ Moderate^b^	
Diarrhoea	No data reported		Not estimable	(0 studies)	**–**	
Adverse outcomes	No data reported		Not estimable	(0 studies)	–	

*CI* confidence interval, *MD* mean difference, *RR* risk ratio

GRADE Working Group grades of evidence

High quality: we are very confident that the true effect lies close to that of the estimate of the effect

Moderate quality: we are moderately confident in the effect estimate: The true effect is likely to be close to the estimate of the effect, but there is a possibility that it is substantially different

Low quality: our confidence in the effect estimate is limited: The true effect may be substantially different from the estimate of the effect

Very low quality: we have very little confidence in the effect estimate: The true effect is likely to be substantially different from the estimate of effect

^a^The risk in the intervention group (and its 95% confidence interval) is based on the assumed risk in the comparison group and the relative effect of the intervention (and its 95% CI)

^b^Rated down for study limitations due to unclear risk of bias across all studies due to lack of blinding of participants, personnel, outcome assessors

^c^Although optimal information size is met, confidence intervals include the null effect as well as appreciable benefit thus rated down for imprecision

^d^Downgraded two levels for very serious imprecision, optimal information size is not met, sample size is 51 participants and only 1 event (< 300)

We did not identify any studies of screening for infection followed by deworming of infected participants.

#### Anaemia

**Fig. 4:**
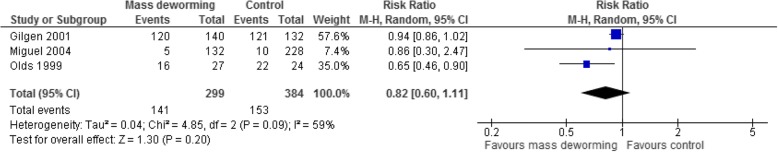
Forest plot for end of study anaemia prevalence as mean difference

There was a pooled relative risk of deworming compared to control of 0.82 (95% CI 0.60 to 1.11; three studies, *n* = 683). The absolute reduction in number of women with anaemia at the end of study was 72 women per 1000 (95% CI from 44 more to 159 fewer) in mass deworming compared to control. This outcome had low-certainty evidence due to high risk of bias and imprecision (Fig. 4).

#### Severe anaemia

Severe anaemia was not reported as an outcome in any of the included studies; however, we were able to calculate the prevalence of severe anaemia in Olds et al. [83] from individual participant data.

**Fig. 5:**
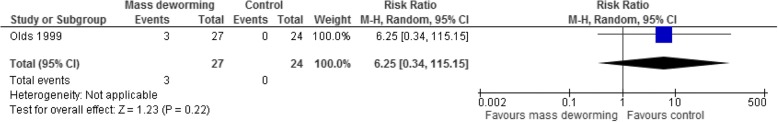
Forest plot for end of study severe anaemia prevalence as mean difference

The number of participants with severe anaemia (haemoglobin levels < 80 g/L) at the end of the study was three in the deworming group compared to zero in the control group, with a relative risk of 6.25 (95% CI 0.34 to 115.15; *n* = 51, one study). This outcome had very low-certainty evidence due to high risk of bias and imprecision because of the few participants [51] and events (Fig. 5).

#### Iron-deficiency

**Fig. 6:**
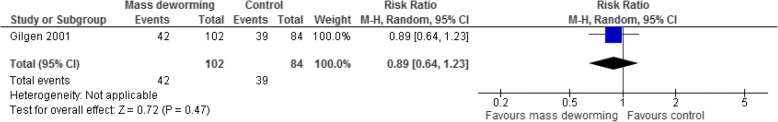
Forest plot for end of study prevalence of iron deficiency as mean difference

We received data on iron-deficiency from Dr. Gilgen [104]. No other studies reported iron-deficiency. A relative risk of 0.89 (95% CI 0.64 to 1.23; *n* = 186, 1 study) was found in the prevalence of iron deficiency (ferritin levels < 12 μg/L) at the end of the study with an absolute risk reduction of 51 women per 1000 (95% CI from 107 more to 167 fewer). This outcome had low-certainty evidence due to high risk of bias and imprecision (Fig. 6).

#### Parasite load

Parasite load (the number of people with soil-transmitted helminthiasis) was reduced with deworming compared to no intervention/placebo in Gilgen et al. [104] and Miguel et al. 2004 [23]. None of the other studies reported parasite load at the end of study.

We found a relative risk of 0.29 (95% CI 0.14 to 0.62; *n* = 1498, 2 studies) for *Ascaris lumbricoides* infection, with an absolute risk reduction of 232 women per 1000 (95% CI from 124 fewer to 281 fewer), in the dewormed group compared to no intervention/placebo.

There was a pooled relative risk of 0.32 (95% CI 0.18 to 0.59; *n* = 1498, 2 studies) for hookworm infection with an absolute risk reduction of 225 women per 1000 (from 136 fewer to 271 fewer), in the dewormed group compared to no intervention/placebo.

For *T*. *trichiura* infection, we found a relative risk of 0.79 (95% CI 0.65 to 0.95; *n* = 1498, 2 studies) with an absolute risk reduction of 64 women per 1000 (from 25 fewer to 97 fewer), in the dewormed group compared to no intervention/placebo.

**Fig. 7:**
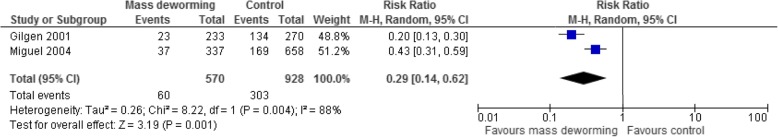
Forest plot for end of study *A. lumbricoides* prevalence as mean difference

**Fig. 8:**
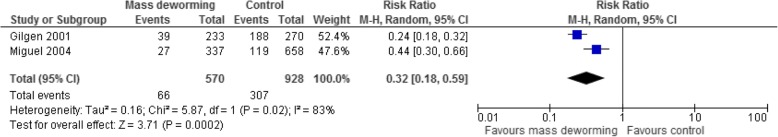
Forest plot for end of study hookworm prevalence as mean difference

**Fig. 9:**
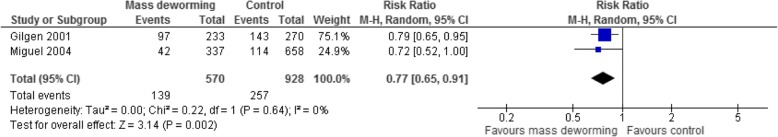
Forest plot for end of study *T. trichiura* prevalence as mean difference

These outcomes were of moderate certainty evidence, downgraded due to risk of bias. These analyses are presented in Figs. 7, 8 and 9.

#### Reinfection

None of the studies reported reinfection rates.

#### Diarrhoea

None of the studies reported the incidence of diarrhoea.

#### All-cause morbidity

None of the studies reported all-cause morbidity for the comparison of deworming to no intervention/placebo.

#### Physical function/work capacity

Physical function and work capacity was reported in the form of labour productivity (kilogrammes of tea leaves plucked per day and wages earned per day) in Gilgen et al. [104]. However, the data provided was not sufficient to conduct an analysis of effect. The study reported no important differences in labour productivity between intervention groups during the trial period.

Physical function/work capacity was not reported in any of the other included studies.

#### Adverse events

Adverse events were not reported in the included studies.

#### Mass deworming with iron compared to iron alone

A separate comparison of deworming + iron vs. iron only was conducted in order to assess the effect of mass deworming when combined with iron supplementation in comparison to iron supplements alone. This analysis found little to no difference between the intervention of deworming + iron compared to iron alone on anaemia prevalence (RR 1.04; 95% CI 0.92 to 1.17, *n* = 266, one study), iron-deficiency prevalence (RR 0.84; 95% CI 0.48 to 1.48, *n* = 187, one study) and all-cause morbidity assessed by conjunctival xerosis (RR 1.00; 95% CI 0.24 to 4.23, *n* = 32, one study).

### Assessment of reporting bias

We did not have enough studies to do funnel plots.

### Subgroup and sensitivity analysis

We did not plan any subgroup analyses as we expected to have insufficient number of studies.

The results for the primary outcomes of anaemia, iron deficiency and infection intensity were not sensitive to restricting to studies with low risk of bias for randomised sequence generation [83, 104].

We did not conduct other planned sensitivity analysis since we did not have outlier studies with very large effects or very precise confidence intervals.

## Discussion

### Summary of main results

We identified four randomised trials that assessed the effects of regular deworming in endemic areas for soil-transmitted helminths in non-pregnant women between the ages of 10 and 49 years.

Mass deworming compared to control probably leads to reduction in ascaris, hookworm and trichuris prevalence, when measured at least 3 months after deworming (moderate quality of evidence). When compared to control, mass deworming may have little or no effect in reducing prevalence of anaemia or iron deficiency in women between the ages of 10 and 49 (low certainty evidence). There is very low certainty evidence that deworming compared to control has an effect on severe anaemia. Work capacity was reported in one study but the effect of deworming was not estimable with the data provided. Diarrhoea, reinfection and adverse outcomes were not reported in any of the included studies.

### Overall completeness and applicability of evidence

These results should be interpreted with caution because of sparse data not meeting the optimal information size for some outcomes, thus important effects on anaemia cannot be ruled out. There was very little evidence available on deworming compared to control for non-pregnant women and adolescent girls, with only four included studies, all of mass deworming. Reinfection, diarrhoea and adverse events were not reported in the included studies. This review highlights the paucity of available studies and evidence for this subpopulation.

Evidence from other populations (children, pregnant women and male and female adults) also suggest little to no effects on anaemia and iron deficiency for deworming alone [28, 106, 107].

### Implications of deviations from the protocol

We were able to include two additional studies which had girls aged 10–12 years [23, 83].

### Quality of the evidence

The four included studies with data available for analysis had unclear or low risk of bias for sequence generation and allocation concealment and were unclear for other domains of bias. We conducted a sensitivity analysis restricted to studies with low risk of bias for sequence generation and the results were in agreement with the overall analysis.

We used the GRADE tool to assess the quality of the available evidence [35]. Quality ranged from moderate to very low across outcomes due to risk of bias and imprecision.

### Potential biases in the review process

We conducted a comprehensive search of electronic databases and trial registers, and supplemented this with searching the included and excluded studies of two published reviews of mass deworming for children [27] and non-pregnant populations [28] which both included grey literature searches. We may have missed studies in adults which were not identified by those reviews or our search strategy.

We did not have sufficient studies to assess publication bias using funnel plots. In addition, disaggregated data was not available for six trials that included this age range. The adoption of the SAGER guidelines on Sex and Gender Equity in Research [108] and the All Trials campaign for open access to trial data may improve the feasibility of these types of analyses in the future.

We limited the interventions to deworming alone. We did not consider concomitant interventions that may have an impact on worm infections such as water, sanitation or hygiene interventions.

### Agreements and disagreements with other studies or reviews

Menstruating girls and women are a high-risk population for anaemia, and previous reviews have either focused on children or on adults or pregnant women, thus not focusing on this priority population. The Cochrane review of mass deworming children for soil-transmitted helminths [106] and a Campbell review of the same question [27] concluded there was little to no effect of mass deworming and screen-to-treat deworming on haemoglobin levels (of about 0.2 g/L difference). The finding of an 18% relative risk reduction in anaemia is also in alignment with a review of non-pregnant populations which included males and females, which found a 13% relative risk reduction in anaemia [28]. Similar results were demonstrated in a Cochrane systematic review of deworming for pregnant women, which found little to no impact of anthelminthics on maternal anaemia in the third trimester (risk ratio 0.94 [95% CI 0.81 to 1.10]) [107]. We did not assess safety in the first trimester of pregnancy. Both Albendazole and Mebendazole are approved for use in the WHO Model Formulary after the first trimester only [109]. Programmes delivering deworming tablets to adolescent girls and women should ensure that those receiving anthelminthic medicines are not pregnant [20, 110].

By bringing together evidence on non-pregnant women and adolescent girls, this review informs policy actions for this population. Since the evidence is so sparse, important effects cannot be ruled out on anaemia.

## Conclusions

### Implications for practice

This review was limited by a paucity of data which cannot exclude important effects of mass deworming on anaemia in non-pregnant adolescent girls and women. Thus, mass deworming may be considered in this population, with the caveats described in the WHO guidelines 2017 and appropriate monitoring for safety and effectiveness, including precaution in ensuring that women and girls receiving anthelminthic medicines are not pregnant.

### Implications for research

Future studies should present sex-disaggregated data and consider populations for whom effects may differ, such as adolescents and those with severe anaemia, and report anaemia and severe anaemia as outcomes.

Implementation research into combined interventions such as deworming with nutritional, environmental, water, sanitation or hygiene interventions may help inform multisector programs that address multiple public health outcomes.

## Additional files

Additional file 1: Review protocol. (DOCX 24 kb)

Additional file 2: PRISMA checklist. (DOC 63 kb)

Additional file 3: Search strategies. (DOCX 24 kb)

Additional file 4: Data extraction form. (XLSX 20 kb)

Additional file 5: Excluded studies. (DOCX 20 kb)

## Abbreviations

CI: Confidence intervals
NR: Not reported
RCT: Randomised controlled trial
STH: Soil-transmitted helminthiasis

## Footnotes

1 Soil-transmitted helminth prevalence is defined as the percentage of population that have one or more soil transmitted helminth infections.
