# Supplementary material for: Deworming in non-pregnant adolescent girls and adult women: a systematic review and meta-analysis
Source: Syst Rev. 2018 Dec 20;7:239. doi: 10.1186/s13643-018-0859-6 (PMC6300900; doi:10.1186/s13643-018-0859-6)
Supplement: Supplementary file 1 — Review protocol. (DOCX 24 kb) [file 13643_2018_859_MOESM1_ESM.docx]

**Additional file 1: Review protocol**

**Deworming for non-pregnant adolescent and adult women**

**Background**

**Description of the condition**

Soil-transmitted helminthiasis (STH) affects 24% (2 billion people) of the global population (WHO, 2015). The burden of disease of STH was estimated at 4.98 million disability adjusted life years DALYS globally in 2010 (Pullan, 2014). These infections rarely cause death, and therefore the burden is predominantly due to morbidity. The most common species of helminths that infect people are the roundworm (*Ascaris lumbricoides*), the whipworm (*Trichuris trichiura*) and hookworms (*Necator americanus* and *Ancylostoma duodenale*). STH has a negative impact on the nutritional status of infected people. These helminths feed on host tissues and blood which results in a loss of iron and protein that can lead to severe iron deficiency, anemia, or other nutritional impairments (WHO, 2015).

**Description of the intervention**

Antihelminthic drugs such as albendazole, mebendazole, levamisole, pyrantel and thiabendazole are administered as pharmacological deworming interventions for patients with soil-transmitted helminth infections. WHO recommends these deworming treatments to be administered periodically to all at-risk populations in areas with endemic STH, with or without previous diagnosis (WHO, 2015).

**How the intervention might work**

Deworming treatments aim to reduce the intensity of helminth infection, to protect infected individuals and prevent further transmission of infection (WHO 2006). Anthelminthic drugs reduce worm burden and in turn can reduce the morbidity associated with STH. With many deworming treatment programs taking place in community-based settings, there is the potential to also see a spill-over effect of decreased worm burden among untreated people and their household members. This occurs because there is a lower risk of untreated people contracting the disease from treated people.

**Why it is important to do this review**

Women of childbearing age are regarded as an at-risk sub-population by the World Health Organization (WHO, 2015). In addition, non-pregnant women deal with monthly blood loss due to menstruation. This can have a detrimental effect on iron levels and anaemia, resulting in a poorer overall nutritional status. Hence, it is important to know whether deworming can improve the nutritional status of menstruating adolescent and adult women.

**Objectives**

This systematic review will evaluate the effects of regular deworming in endemic areas for soil-transmitted helminths in non-pregnant women between the ages of 12 and 49 years globally on anemia prevalence, iron deficiency, parasite load, diarrhea prevalence, reinfection and all-cause morbidity.

**Methods**

**Criteria for considering studies for this review**

***Types of studies***

We will include randomised controlled trials, which may be randomised at the individual or cluster level. We will also include quasi-experimental studies such as controlled before and after studies and interrupted time series (with at least three times points before and after the intervention, with or without a control group). We will also include studies with a post-only measurement, providing the baseline groups are considered comparable on potential confounders such as worm prevalence, education level and anemia prevalence.

***Types of participants***

- Non-pregnant women between the ages of and including 12 and 49.

We will include studies that report results for women in the above age groups, disaggregated from other populations. If studies include this population, but data is not reported separately from women aged 12-49 years, then we will identify this as a potentially eligible study. However, we will not be able to request additional data before March 31, 2015.

***Types of interventions***

- Deworming treatment targeted at soil-transmitted helminth infections: albendazole, mebendazole, pyrantel, piperazine, levamisole, thiabendazole
- Compared to no intervention or a placebo

The Deworming WHO Model List of Essential Medicines was consulted to select the above listed deworming drugs (WHO Medicines, 2015). Any other drug for STH will also be included.

Other interventions will be excluded unless present in both treatment and control arms. For example, concomitant iron will be allowed as long as it is provided in both the treatment and control groups.

***Types of outcome measures***

The outcomes outlined below were selected based on ratings made by the WHO guideline panel for the review.

Primary outcomes

- Anaemia (defined as haemoglobin concentration of less than 120 g/L for non-pregnant women, adjusted by altitude where appropriate)
- Iron deficiency (as defined by using ferritin concentrations < 15 μg/L)
- Parasite load (egg count/g of faeces)
- Diarrhoea (three liquid stools or more per day)
- Severe anaemia (defined as haemoglobin concentration lower than 70 g/L, adjusted by altitude where appropriate)
- Reinfection
- All-cause morbidity (number of patients with at least one episode of any disease during the study period)

Secondary outcomes

- Physical function/work capacity
- Adverse events (any, as defined by trialists)

This outcome was discussed by the WHO guideline development group but not scored as critical for decision-making, hence it will be assessed as a secondary outcome.

**Search methods for identification of studies**

***Electronic searches***

The search includes the following health and non-health electronic databases: MEDLINE, CINAHL, LILACS, EMBASE, the Cochrane Library, Econlit, Internet Documents in Economics Access Service (IDEAS), Public Affairs Information Service (PAIS), Social Services Abstracts, Global Health CABI and CAB Abstracts.

***Searching other resources***

We will not conduct a search of grey literature or use reference lists from previous systematic reviews, to identify potentially relevant studies. We will also not contact authors for additional details (e.g. disaggregated data).

**Data collection and analysis**

***Selection of studies***

Two reviewers will independently screen titles and abstracts based on the following questions: a) does the intervention include pharmacologic deworming treatment which is provided by mass or targeted administration to an identified high-risk group? b) is at least one of: anemia, iron deficiency, parasite load, diarrhoea, severe anemia, reinfection, all-cause morbidity in the outcomes measured? c) does the population include women between the ages of 12 and 49 years ? d) is the length of time from intervention to follow-up four months or longer? and e) does the study design include an appropriate comparison group (i.e., control group or pre-post or post-only if baseline characteristics similar)?

We will pre-test the title and abstracts screening questions. If any one of these questions is answered as ‘no’, then the study will be excluded from further consideration. If all questions are answered as ‘yes’, then the study will be included for full-text screening. Coding for screening will be entered into systematic review software manager, Covidence. After each reviewer has independently screened studies, any discrepancies around decisions for inclusion or exclusion will be discussed and reconciled accordingly. Full text will be retrieved for titles and abstracts accepted for inclusion after discussion by both reviewers. The full text will be screened by two reviewers for inclusion according to the pre-specified eligibility criteria. Any disagreements will be settled by discussion with a third party who will review the full text and decide whether it meets the inclusion criteria. For judgments related to appropriate control for confounders, we will consult with a statistician (AH).

***Data extraction and management***

The two reviewers will conduct independent data extraction and risk of bias assessment of all included studies. The data extraction form will model the EPOC data collection form. We will pre-test the data extraction form. Information to be extracted includes data on study design, statistical analysis, details about the participants (including the number in each group), setting (e.g. endemicity, sanitation), intervention (e.g. type of drugs, dose, frequency and process of implementation), comparison, cost-effectiveness, outcomes (including whether outcomes are validated). We will extract process data on the implementation of the intervention such as method of delivering deworming (e.g. provision of deworming integrated with other programs), amount of supervision. Where possible, we will extract data about socio-demographic variables associated with disadvantage, across factors described by the acronym PROGRESS (Place of residence, Race/ethnicity, Occupation, Gender/sex, Religion, Education, Socioeconomic status and Social capital) (Tugwell 2006). We will extract data on any effect modifier analyses (e.g. subgroup analyses and meta-regression) conducted in the primary studies. We will compare the extraction by both reviewers, and reach consensus by discussion and consultation with a third reviewer, if necessary.

***Assessment of risk of bias in included studies***

We will use the Cochrane Risk of Bias tool to assess potential sources of bias in the included randomized controlled studies (Cochrane Handbook, 2011). The main categories of bias that will be assessed are: selection bias, performance bias, detection bias, attrition bias and outcome reporting bias.

Since we will also include controlled before-after studies, interrupted time series and post-only studies, we will use EPOC's suggested risk of bias criteria. For controlled before-after studies, we will additionally assess baseline imbalance, similarity of outcome measurements, and the level of protection against contamination in each study. For interrupted time series studies, we will also assess the dependence of the intervention on other changes, pre-specification of the shape of the intervention, and the likelihood of the intervention to affect data collection (EPOC, 2015)

Risk of bias will be assessed for each outcome in each study.

***Measures of treatment effect***

The effect size of the continuous outcomes will be analyzed as weighted mean differences of change scores as these will be measured using the same units across studies. Standard deviations for each effect estimate will also be calculated. Dichotomous outcomes will be analyzed as relative risks, using random-effects methods. We will use a random-effects model since we expect the underlying treatment effect will vary depending on the context, populations and setting. We will report analyses for each outcome and follow-up period separately. We will not conduct network meta-analyses for any of the outcomes.

***Unit of analysis issues***

Where the unit of allocation is by groups (e.g. schools, communities, village, region), we will use the standard deviation adjusted for clustering, if provided by the study. In the case of randomised studies, if the study has not adjusted for clustering, we will adjust the standard deviations using the variance inflation factor, as described in the Cochrane Handbook (Cochrane Handbook, 2011).The variance inflation factor is calculated using the equation: (1 + (m-1) x ICC) where (m) is the cluster size and ICC is the intra cluster correlation. If cluster size is not reported, the number of participants in each analysis or total number of participants (if former not available) will be divided by the number of clusters to calculate cluster size. If ICC is not reported, we will estimate ICC values for the corresponding outcome measure using published ICCs for similar outcome measures. The effect of ICC values will be assessed using sensitivity analysis. For quasi-randomised studies, where an adjusted estimate has been determined but clustering is not taken into consideration, we will derive procedures to accommodate for clustering in the modelling process, if possible.

***Dealing with missing data***

We will attempt to collect complete data on items in the data extraction form. If standard deviation or standard error is not provided, we will calculate it from other information provided such as exact p-values, F-tests or ranges, using formulae in the Cochrane Handbook. Information will not be sought from the authors of the study if information reported is insufficient to calculate effect size and standard deviation. We will not impute missing values (e.g. missing variance or outcome data).

***Assessment of heterogeneity***

Heterogeneity will be assessed by visual inspection of forest plots, chi-squared test and I2. I2 will be used to quantify inconsistency across studies, as it describes the percentage of variability in effect estimates that is due to heterogeneity. We will explore heterogeneity using meta-regression, subgroup and sensitivity analyses, as described below.

***Assessment of reporting biases***

Since we are not conducting a grey literature search, we may miss unpublished studies which could result in reporting biases. We will assess this using a funnel plot, if we find >10 studies.

***Data synthesis***

The magnitude of effect and quality of evidence will be presented clearly and concisely in a summary of findings table, with the seven primary outcomes listed above, and determined by the guideline expert panel. Quality will be assessed by the GRADE criteria for this summary of findings table, using GRADEpro software.

***Subgroup analysis and investigation of heterogeneity***

We will conduct the following subgroup analyses to explore heterogeneity:

- By baseline prevalence of any soil-transmitted helminth in the trial, using the cutoffs from the WHO guidelines (WHO Guideline, 2011): less than 20%, 20 to 49%, 50% or higher, unknown/not reported
- By anaemia prevalence in the study: anaemic (hemoglobin <120 g/L), non-anaemic, mixed/not reported. Classification of anemia prevalence according to proportion of anemic cases in population: severe = ≥40%; moderate = 20.0-39.9%; mild = 5.0-19.9%; normal = ≤ 4.9% (WHO, 2011).
- By intensity of infection (WHO, 2002):

1. Light-intensity infections: 1-4999 eggs per gram of feces (epg) in *A. lumbricoides*; 1-999 epg in *T. trichiura*; 1-1999 epg in hookworms
2. Moderate-intensity infections: 5000-49999 epg in *A. lumbricoides*; 1000-9999 epg in *T. trichiura*; 2000-3999 epg in hookworms
3. Heavy-intensity infections: ≥50000 epg in *A. lumbricoides*; ≥10000 epg in *T. trichiura*; ≥4000 epg in hookworms

- By screening: yes/no (i.e. studies with screening as an eligibility criteria such that the entire study population is infected)
- By eligibility criteria that restricts to less severely affected groups: we will compare studies which exclude severely affected women from participating (e.g. by referring to treatment) to those which accept "all-comers".

***Sensitivity analysis***

Sensitivity analyses will be conducted to assess the impact of outlier individual studies (e.g. very large studies, very large effects, very precise confidence intervals) on the overall effect size. We will also assess effects of risk of bias, treatment compliance and imputed variance inflation factor using sensitivity analyses.
