# Supplementary material for: Deworming in non-pregnant adolescent girls and adult women: a systematic review and meta-analysis
Source: Syst Rev. 2018 Dec 20;7:239. doi: 10.1186/s13643-018-0859-6 (PMC6300900; doi:10.1186/s13643-018-0859-6)
Supplement: Supplementary file 3 — Search strategies. (DOCX 24 kb) [file 13643_2018_859_MOESM3_ESM.docx]

**Additional file 3: Search strategies**

**C1 - Database: Ovid MEDLINE(R) and Epub Ahead of Print, In-Process & Other Non-Indexed Citations and Daily <1946 to September 21, 2018>
Search Strategy:**--------------------------------------------------------------------------------

1 exp Helminthiasis/ (121358)
2 exp Central Nervous System Helminthiasis/ (2047)
3 (helminthias#s or bertiellias#s or cenurias#s or coenurias#s or dipylidias#s or raillietinias#s or Echinococcos#s or hydatidos#s or hydatid cyst$ or Diphyllobothrias#s or Hymenolepias#s or Moniezias#s or Taenias#s or Sparganos#s or Cysticercos#s or Neurocysticercos#s or Trichinellos#s or Trichurias#s or Anisakias#s or Ascarias#s or Ascaridiasi#s or toxascariasi#s or Toxocarias#s or Clonorchias#s or Dicrocoelias#s or Echinostomias#s or Fasciolias#s or Fascioloidias#s or Opisthorchias#s or Paragonimias#s or ortrichinellos#s or Trichostrongyloidias#s or ancylostomias#s or enterobias#s or Oesophagostomias#s).tw. (33136)
4 ((Helminth$ or tapeworm or nematomorpha or Cestode or Nematode or hymenolepis or Platyhelminth$ or taenia or Adenophorea or aphasmidia or Enoplida or Larva Migrans or dew itch$ or creeping eruption$ or Secernentea or Ascaridida or Oxyurida or Rhabditida or Spirurida or Strongylida or whipworm$ or whip worm$ or hookworm$ or hook worm$ or roundworm$ or round worm$ or geohelminth$ or flukes americanus or Enterobius or Oxyuroidea or Oxyurida or Trichuris or Trichuroidea or Capillaria or Trichinella or Strongyloid$ or Oesophagostomum or Strongylus or Acanthocephala or Moniliformis or Adenophorea or Enoplida or Secernentea or Ascaridida or Rhabditida or Nematoda or Cestoda or Trematod$ or Turbellaria or Platyhelminth$ or Rotifera or trematode$ or ascarid$ or Toxocara$ or ancylostoma$ or Necator$ or Ascaris or Ascaridida or Ancylostoma) adj2 infection$).tw. (12331)
5 or/1-4 (128503)
6 Albendazole/ (4071)
7 Mebendazole/ (1855)
8 exp Piperazines/ (70750)
9 Levamisole/ (4237)
10 exp Pyrantel/ (581)
11 Ivermectin/ (5771)
12 exp Anthelmintics/ (56299)
13 (Ivermectin or Albendazole or Mebendazole or Piperazine$ or Levamisole or Pyrantel or thiabendazole or Anthelmint$ or anti-helmint$ or deworm$ or de-worm$ or vermifug$).tw. (30274)
14 or/6-13 (137854)
15 5 and 14 (24961)
16 animal/ (6268767)
17 human/ (17288381)
18 16 not (16 and 17) (4464151)
19 15 not 18 (15695)
20 adolescent/ or exp child/ or young adult/ or adult/ (6086699)
21 (child* or paediatric* or pediatric* or youth or adolescen* or teen* or adult*).tw. (2426910)
22 19 and (20 or 21) (9409)
23 (female or girl or girl$ or wom#n).tw. (1677259)
24 female/ (8038412)
25 22 and (23 or 24) (5788)
26 (clinical trial or randomized controlled trial).pt. (766209)
27 (randomi#ed or placebo).mp. (841910)
28 (pre test or pretest or (posttest or post test)).tw. (24334)
29 (time adj series).tw. (25392)
30 (controlled adj before).tw. (1094)
31 or/26-30 (1157566)
32 25 and 31 (843)
33 meta-analysis.mp,pt. (149064)
34 cochrane database of systematic reviews.jn. (13805)
35 (systematic review or systematic literature review).tw. (119759)
36 (meta synthesis or meta synthesis or integrative review or integrative research review or rapid review).tw. (3324)
37 or/33-36 (226953)
38 25 and 37 (25)
39 32 or 38 (853)
40 limit 39 to ed=20170210-20180925 (50)

**C1 - Database: Embase Classic+Embase <1947 to 2018 September 21>
Search Strategy:**--------------------------------------------------------------------------------
1 exp Helminthiasis/ (127200)
2 central nervous system infection/ (8306)
3 (helminthias#s or bertiellias#s or cenurias#s or coenurias#s or dipylidias#s or raillietinias#s or Echinococcos#s or hydatidos#s or hydatid cyst$ or Diphyllobothrias#s or Hymenolepias#s or Moniezias#s or Taenias#s or Sparganos#s or Cysticercos#s or Neurocysticercos#s or Trichinellos#s or Trichurias#s or Anisakias#s or Ascarias#s or Ascaridiasi#s or toxascariasi#s or Toxocarias#s or Clonorchias#s or Dicrocoelias#s or Echinostomias#s or Fasciolias#s or Fascioloidias#s or Opisthorchias#s or Paragonimias#s or ortrichinellos#s or Trichostrongyloidias#s or ancylostomias#s or enterobias#s or Oesophagostomias#s).tw. (39426)
4 ((Helminth$ or tapeworm or nematomorpha or Cestode or Nematode or hymenolepis or Platyhelminth$ or taenia or Adenophorea or aphasmidia or Enoplida or Larva Migrans or dew itch$ or creeping eruption$ or Secernentea or Ascaridida or Oxyurida or Rhabditida or Spirurida or Strongylida or whipworm$ or whip worm$ or hookworm$ or hook worm$ or roundworm$ or round worm$ or geohelminth$ or flukes americanus or Enterobius or Oxyuroidea or Oxyurida or Trichuris or Trichuroidea or Capillaria or Trichinella or Strongyloid$ or Oesophagostomum or Strongylus or Acanthocephala or Moniliformis or Adenophorea or Enoplida or Secernentea or Ascaridida or Rhabditida or Nematoda or Cestoda or Trematod$ or Turbellaria or Platyhelminth$ or Rotifera or trematode$ or ascarid$ or Toxocara$ or ancylostoma$ or Necator$ or Ascaris or Ascaridida or Ancylostoma) adj2 infection$).tw. (14532)
5 or/1-4 (143610)
6 Albendazole/ (13135)
7 Mebendazole/ (5839)
8 exp piperazine derivative/ (419958)
9 Levamisole/ (11610)
10 exp Pyrantel/ (704)
11 Ivermectin/ (10773)
12 exp anthelmintic agent/ (125899)
13 (Ivermectin or Albendazole or Mebendazole or Piperazine$ or Levamisole or Pyrantel or thiabendazole or Anthelmint$ or anti-helmint$ or deworm$ or de-worm$ or vermifug$).tw. (37626)
14 or/6-13 (542797)
15 5 and 14 (35259)
16 animal/ (1869055)
17 human/ (19942210)
18 16 not (16 and 17) (1421994)
19 15 not 18 (32179)
20 adolescent/ or exp child/ or young adult/ or adult/ (8626180)
21 (child* or paediatric* or pediatric* or youth or adolescen* or teen* or adult*).tw. (3261180)
22 19 and (20 or 21) (16328)
23 (female or girl or girl$ or wom#n).tw. (2448751)
24 female/ (8338994)
25 22 and (23 or 24) (8573)
26 meta-analys$.mp. or search$.tw. or review.pt. (2855599)
27 cochrane database of systematic reviews.jn. (12658)
28 (systematic review or systematic literature review).tw. (146716)
29 (random$ or double-blind$).tw. or placebo$.mp. (1594835)
30 (pre test or pretest or (posttest or post test)).tw. (33968)
31 (time adj series).tw. (28175)
32 (controlled adj before).tw. (1294)
33 or/26-28 (2878480)
34 or/29-32 (1647066)
35 25 and 33 (350)
36 25 and 34 (765)
37 35 or 36 (1080)
38 limit 37 to medline (128)
39 37 not 38 (952)
40 limit 39 to yr="2017- 2018" (148)

**C1 - Database: EBM Reviews - Cochrane Central Register of Controlled Trials <August 2018>, EBM Reviews - Cochrane Database of Systematic Reviews <2005 to September 19, 2018>
Search Strategy:**--------------------------------------------------------------------------------
1 exp Helminthiasis/ (1196)
2 exp Central Nervous System Helminthiasis/ (44)
3 (helminthias#s or bertiellias#s or cenurias#s or coenurias#s or dipylidias#s or raillietinias#s or Echinococcos#s or hydatidos#s or hydatid cyst$ or Diphyllobothrias#s or Hymenolepias#s or Moniezias#s or Taenias#s or Sparganos#s or Cysticercos#s or Neurocysticercos#s or Trichinellos#s or Trichurias#s or Anisakias#s or Ascarias#s or Ascaridiasi#s or toxascariasi#s or Toxocarias#s or Clonorchias#s or Dicrocoelias#s or Echinostomias#s or Fasciolias#s or Fascioloidias#s or Opisthorchias#s or Paragonimias#s or ortrichinellos#s or Trichostrongyloidias#s or ancylostomias#s or enterobias#s or Oesophagostomias#s).tw. (444)
4 ((Helminth$ or tapeworm or nematomorpha or Cestode or Nematode or hymenolepis or Platyhelminth$ or taenia or Adenophorea or aphasmidia or Enoplida or Larva Migrans or dew itch$ or creeping eruption$ or Secernentea or Ascaridida or Oxyurida or Rhabditida or Spirurida or Strongylida or whipworm$ or whip worm$ or hookworm$ or hook worm$ or roundworm$ or round worm$ or geohelminth$ or flukes americanus or Enterobius or Oxyuroidea or Oxyurida or Trichuris or Trichuroidea or Capillaria or Trichinella or Strongyloid$ or Oesophagostomum or Strongylus or Acanthocephala or Moniliformis or Adenophorea or Enoplida or Secernentea or Ascaridida or Rhabditida or Nematoda or Cestoda or Trematod$ or Turbellaria or Platyhelminth$ or Rotifera or trematode$ or ascarid$ or Toxocara$ or ancylostoma$ or Necator$ or Ascaris or Ascaridida or Ancylostoma) adj2 infection$).tw. (513)
5 or/1-4 (1626)
6 Albendazole/ (389)
7 Mebendazole/ (122)
8 exp Piperazines/ (5715)
9 Levamisole/ (354)
10 exp Pyrantel/ (35)
11 Ivermectin/ (279)
12 exp Anthelmintics/ (1502)
13 (Ivermectin or Albendazole or Mebendazole or Piperazine$ or Levamisole or Pyrantel or thiabendazole or Anthelmint$ or anti-helmint$ or deworm$ or de-worm$ or vermifug$).tw. (2116)
14 or/6-13 (8205)
15 5 and 14 (1184)
16 animal/ (9404)
17 human/ (109)
18 16 not (16 and 17) (9404)
19 15 not 18 (774)
20 adolescent/ or exp child/ or young adult/ or adult/ (354228)
21 (child* or paediatric* or pediatric* or youth or adolescen* or teen* or adult*).tw. (195974)
22 19 and (20 or 21) (589)
23 (female or girl or girl$ or wom#n).tw. (146798)
24 female/ (415036)
25 22 and (23 or 24) (353)

**C1 - Database: Food Science and Technology Abstracts <1969 to 2018 September Week 3>
Search Strategy:**--------------------------------------------------------------------------------
1 (Helminth$ or tapeworm or nematomorpha or Cestode or Nematode or hymenolepis or Platyhelminth$ or taenia or Adenophorea or aphasmidia or Enoplida or Larva Migrans or dew itch$ or creeping eruption$ or Secernentea or Ascaridida or Oxyurida or Rhabditida or Spirurida or Strongylida or whipworm$ or whip worm$ or hookworm$ or hook worm$ or roundworm$ or round worm$ or geohelminth$ or flukes americanus or Enterobius or Oxyuroidea or Oxyurida or Trichuris or Trichuroidea or Capillaria or Trichinella or Strongyloid$ or Oesophagostomum or Strongylus or Acanthocephala or Moniliformis or Adenophorea or Enoplida or Secernentea or Ascaridida or Rhabditida or Nematoda or Cestoda or Trematod$ or Turbellaria or Platyhelminth$ or Rotifera or trematode$ or ascarid$ or Toxocara$ or ancylostoma$ or Necator$ or Ascaris or Ascaridida or Ancylostoma).tw. (1529)
2 (helminthias#s or bertiellias#s or cenurias#s or coenurias#s or dipylidias#s or raillietinias#s or Echinococcos#s or hydatidos#s or hydatid cyst$ or Diphyllobothrias#s or Hymenolepias#s or Moniezias#s or Taenias#s or Sparganos#s or Cysticercos#s or Neurocysticercos#s or Trichinellos#s or Trichurias#s or Anisakias#s or Ascarias#s or Ascaridiasi#s or toxascariasi#s or Toxocarias#s or Clonorchias#s or Dicrocoelias#s or Echinostomias#s or Fasciolias#s or Fascioloidias#s or Opisthorchias#s or Paragonimias#s or ortrichinellos#s or Trichostrongyloidias#s or ancylostomias#s or enterobias#s or Oesophagostomias#s).tw. (439)
3 helminths/ (74)
4 exp parasites/ (3283)
5 or/1-4 (4052)
6 exp anthelmintics/ (216)
7 (Ivermectin or Albendazole or Mebendazole or Piperazine$ or Levamisole or Pyrantel or thiabendazole or Anthelmint$ or anti-helmint$ or deworm$ or de-worm$ or vermifug$).tw. (1484)
8 6 or 7 (1509)
9 5 and 8 (75)
10 limit 9 to yr="2017- 2018" (4)

**Interface - EBSCOhost Research Databases, Database - CINAHL
Monday, September 24, 2018 1:04:55 PM**

| **#** | **Query** | **Results** |
| --- | --- | --- |
| S7 | (S3 AND S6) | 270 |
| S6 | (S4 OR S5) | 977 |
| S5 | TX (Ivermectin or Albendazole or Mebendazole or Piperazine$ or Levamisole or Pyrantel or thiabendazole or Anthelmint$ or anti-helmint$ or deworm$ or de-worm$ or vermifug$)) | 514 |
| S4 | MH “Anthelmintics+” | 593 |
| S3 | (S1 OR S2) | 1,421 |
| S2 | TX helminthias#s or bertiellias#s or cenurias#s or coenurias#s or dipylidias#s or raillietinias#s or Echinococcos#s or hydatidos#s or hydatid cyst$ or Diphyllobothrias#s or Hymenolepias#s or Moniezias#s or Taenias#s or Sparganos#s or Cysticercos#s or Neurocysticercos#s or Trichinellos#s or Trichurias#s or Anisakias#s or Ascarias#s or Ascaridiasi#s or toxascariasi#s or Toxocarias#s or Clonorchias#s or Dicrocoelias#s or Echinostomias#s or Fasciolias#s or Fascioloidias#s or Opisthorchias#s or Paragonimias#s or ortrichinellos#s or Trichostrongyloidias#s or ancylostomias#s or enterobias#s or Oesophagostomias#s | 1,421 |
| S1 | MH Helminthiasis | 391 |
