# Supplementary material for: Deworming in non-pregnant adolescent girls and adult women: a systematic review and meta-analysis
Source: Syst Rev. 2018 Dec 20;7:239. doi: 10.1186/s13643-018-0859-6 (PMC6300900; doi:10.1186/s13643-018-0859-6)
Supplement: Supplementary file 5 — Excluded studies. (DOCX 20 kb) [file 13643_2018_859_MOESM5_ESM.docx]

**Additional file 5: Excluded studies**

| **Author, year** | **Reason for exclusion** |
| --- | --- |
| Adams, 1991 | Duration <4 months |
| Adams, 2004 | No relevant outcomes |
| Albonico, 1999 | No relevant outcomes |
| Araujo, 1987 | Mixed intervention, no appropriate control group |
| Azomahou, 2012 | No relevant outcomes |
| Awasthi, 2000 | Children <10 years |
| Beach, 1999 | No relevant outcomes |
| Beasley, 1999 | Mixed intervention, no appropriate control group |
| Belkind-Valdovinos 2003 | No relevant outcomes |
| Bhargava, 2003 | Mixed intervention, no appropriate control group |
| Biggelaar 2004 | No relevant outcomes |
| Boivin 1993 | Duration <4 months |
| Brabin, 1992 | Not a study |
| Casey, 2009 | Mixed intervention, no appropriate control group |
| Cervoni, 1971 | Duration <4 months |
| Chopra, 2006 | No relevant outcomes |
| Clarke 2018 | Mixed intervention, no appropriate control group |
| Cleary, 2007 | No relevant outcomes |
| Cooper, 2006 | No relevant outcomes |
| Dossa, 2001 | Children <10 years |
| Dunn, 1955 | No relevant outcomes |
| Ebenezer, 2013 | Children <10 years |
| Forrester, 1998 | No relevant outcomes |
| Friis, 2003 | Mixed intervention, no appropriate control group |
| Garg, 2002 | Children <10 years |
| Garner, 2013 | Children <10 years |
| Goldsmid, 1973 | No relevant outcomes |
| Goodwin, 1958 | No relevant outcomes |
| Goto, 2009 | Children <10 years |
| Grigorenko, 2006 | No relevant outcomes |
| Guyatt, 2001 | Mixed intervention, no appropriate control group |
| Gyorkos, 2013 | No relevant outcomes |
| Kamble, 2011 | No appropriate control group |
| Kinung’hi, 2015 | No appropriate control group |
| Kepha, 2014 | No relevant outcomes |
| Kirwan, 2010 | Children <10 years |
| Kloetzel, 1982 | Children <10 years |
| Koukounari, 2006 | Mixed intervention, no appropriate control group |
| Koukounari, 2007 | Mixed intervention, no appropriate control group |
| Kruger, 1996 | Children <10 years |
| Kvalsvig, 1991 | No relevant outcomes |
| Le Huong, 2007 | Children <10 years |
| Lynch, 1993 | No relevant outcomes |
| Masoda, 2016 | No relevant outcomes |
| Mofid 2017 | Children <10 years |
| Moser 2017 | Mixed intervention, no appropriate control group |
| Mpoya, 2015 | No appropriate control group |
| Mwaniki, 2002 | No relevant outcomes |
| Ndibazza, 2012 | Children <10 years |
| Nga, 2009 | Children <10 years |
| Nga, 2011 | Children <10 years |
| Olsen, 2003 | No relevant outcomes |
| Ostwald, 1984 | Children <10 years |
| Passerini, 2012 | No relevant outcomes |
| Pond, 1970 | No relevant outcomes |
| Rohner, 2010 | Mixed intervention, no appropriate control group |
| Sargent, 1974 | Duration <4 months |
| Stoltzfus, 2004 | Children <10 years |
| Taylor, 2001 | Mixed intervention, no appropriate control group |
| Wang, 1987 | No relevant outcomes |
| Wiria, 2013 | No relevant outcomes |

**Studies with disaggregated data unavailable**

| **Author, year** | **Reason for exclusion** | |
| --- | --- | --- |
| Bhoite, 2012 | Disaggregated data unavailable | |
| Barda 2018 | Disaggregated data unavailable | |
| Liu, 2017 | Disaggregated data unavailable | |
| DeRuiter 2017 | Disaggregated data unavailable |  |
| Stoltzfus, 1998 | Disaggregated data unavailable | |
| Yap, 2014 | Disaggregated data unavailable | |
